# Epstein-Barr Virus-Positive Not Otherwise Specified (EBV+ NOS) Lymphoma Presentation of Primary Bone Tumor Underlying a Pathological Fracture

**DOI:** 10.7759/cureus.26340

**Published:** 2022-06-26

**Authors:** Abhishek Tippabhatla, Rediet T Atalay, Alex Gyftopoulos, Girma M Ayele, Miriam B Michael

**Affiliations:** 1 Orthopedics, Howard University College of Medicine, Washington, D.C., USA; 2 Internal Medicine, Howard University College of Medicine, Washington, D.C., USA; 3 Internal Medicine, University of Maryland Medical Center, Baltimore, USA

**Keywords:** ebv-positive, primary bone tumor, bone lymphoma, pathologic fracture, bone tumor

## Abstract

Diffuse large B-cell lymphoma (DLBCL) is the most common non-Hodgkin's lymphoma (NHL) and accounts for approximately 25% of all NHLs in developed countries. The patients usually present with constitutional symptoms and rapidly enlarging lymphadenopathy and symptomatic mass typically located in the neck or abdomen, along with an aggressive disease course. Most of the patients present with advanced disease with 60% presenting with stage 3 or 4, and those who present with extranodal involvement are usually seen at an earlier stage. Different conditions are associated with non-Hodgkin’s lymphoma ranging from hereditary immunodeficiency disorders, autoimmune disorders, infections such as HIV, Epstein-Barr virus (EBV), hepatitis C virus (HCV), *Helicobacter pylori*, and drugs such as immunosuppressants and chemotherapeutic agents. Epstein-Barr virus (EBV) is the main etiology of DLBCLs with an identified cause and it accounts for 10% of all DLBCLs.

We report a case of a 51-year-old woman who came with a non-traumatic left femur fracture and was subsequently found to have EBV-positive DLBCL. Lymphoma commonly presents as a lymph node swelling and it’s uncommon to present as primary bone disease.

## Introduction

Human herpesvirus 4, commonly known as the Epstein-Barr virus (EBV), is a highly prevalent double-stranded DNA virus associated with a wide range of lymphoid malignancies, such as Burkitt’s lymphoma and B-cell lymphoproliferative diseases and Hodgkin's lymphoma. It is linked to the development of 1-2% of all cancers worldwide. EBV causes various structural and epigenetic alterations when inducing malignancies; EBV-encoded gene products and microRNAs modify the tumor growth due to its effect on the host immunity [1].

EBV-positive diffuse large B-cell lymphoma (DLBCL) found to be more common in the Asian population (8-11%) compared to the Western population (<5%) [2]. Meta-analysis on EBV+DLBCL demonstrates the rarity of this disease, with a pooled proportion of only 7.9% among de novo DLBCL cases [3].

## Case presentation

A 51-year-old female presented with a non-traumatic left femur fracture. She has known attention deficit hyperactivity disorder (ADHD) currently maintained on amphetamine-dextroamphetamine. She presented with sudden sharp pain and an audible snap in her left thigh which made her unable to move while she was working in the garden. She presented to the ER with significant pain in her left thigh and was unable to bear weight. On presentation, she admitted that she had some dull left thigh pain for about five months and she has been managing it conservatively with over-the-counter medications. She denied weight loss, fever, dyspnea on exertion, chest pain, nausea, vomiting, or rash. She has a remote history of tobacco use 20 years ago and occasionally vapes. 

**Figure 1:**
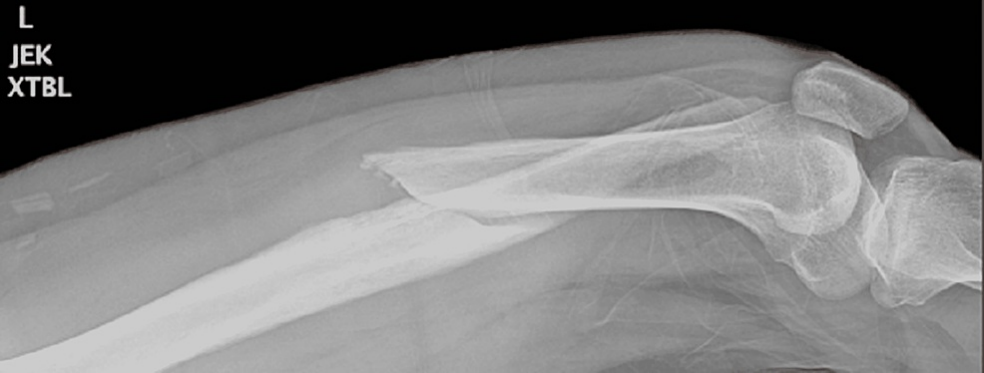
Femur x-ray showing the distal displaced femoral fracture (view 1)

**Figure 2:**
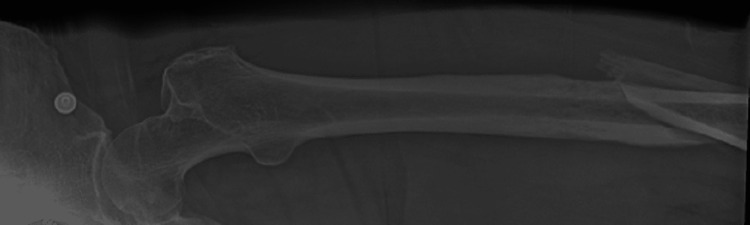
Femur x-ray showing the distal displaced femoral fracture (view 2)

On examination, her vital signs were stable. Physical examination was significant for swelling and tenderness of the left thigh. The left femur x-ray showed a displaced distal femur fracture (Figures 1, 2). The whole-body scan came back negative. Left femur lesion biopsy showed an EBV-positive diffuse large B-cell lymphoma-not otherwise specified (EBV+DLBCL-NOS). Subsequent fracture stabilization and open reduction and internal fixation (ORIF) were performed. The patient was subsequently appointed to an oncologist for staging and subsequent chemotherapy and radiation.

**Figure 3:**
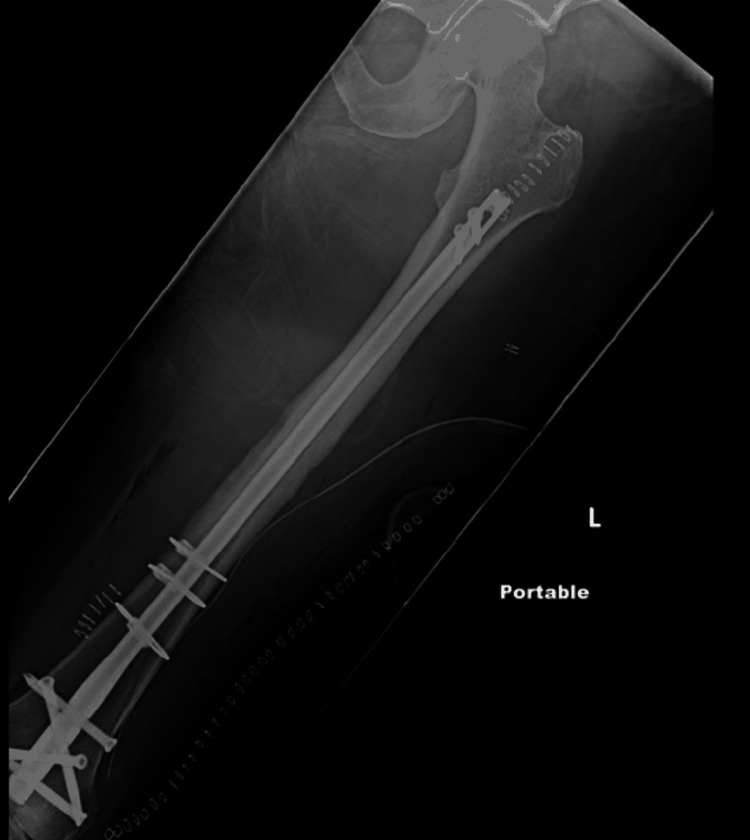
Postoperative open reduction and internal fixation (ORIF) x-ray showing good fixation

Labs at presentation were unremarkable with a WBC count of 7k, RBC of 10.9, and platelets of 316k. Electrophoresis and immunofixation studies are negative. Postoperative follow-up was uneventful, the patient was ambulating well except for mild pain in the left leg and a postoperative x-ray showing good fixation (Figure 3). 

## Discussion

EBV infection is a very common infection affecting most people in the world. Infection occurring in adults can cause infectious mononucleosis even though most infected are children. EBV causes an infection that stays dormant in the B cells due to its unique genes that activate growth. The host immune system, specifically T cells, controls the growth of the B cells that are infected with EBV. Most of the individuals that have been infected with EBV remain asymptomatic but some may develop malignancies that are associated with EBV like Hodgkin's lymphoma, Burkitt's lymphoma, T-cell lymphoma, and B-cell lymphoproliferative disease [4]. 

It remains recognized that 10% of DLBCL are EBV-positive. The classic demographic of EBV+DLBCL-NOS predominantly affects patients >50 years of age, similar to our case [2]. Yet, the absence of any comorbidities and presence of clinical stability render this case unique. Oftentimes, EBV+DLBCL-NOS is a lymphoma subtype that presents in patients with no underlying immunodeficiency [5]. This case is unique in regards to the development of a pathological fracture secondary to primary lymphoma of bone, which is exceedingly rare.

Primary lymphoma of bone is usually DLBCL with primary bone involvement and no extranodal associations; this malignancy involves the appendicular skeleton in 37% of all cases [6]. Twenty-five percent of patients with primary lymphoma of bone may present with pathological fractures on presentation, and femur involvement is most commonly evidenced similar to our case [7]. Yet, malignant involvement primarily of the bone is rare, and even among cases of primary lymphoma of bone involving the long bones, pathological fractures are very rare. In a large study reporting 131 patients diagnosed with primary lymphoma of bone, only nine patients incurred pathological fractures [8]. In total, only 11 cases are reported in the literature that involve pathological fractures secondary to primary lymphoma of bone, an event that is noticeably rare [9,7].

It was previously thought that DLBCL patients over 50 years of age with EBV positivity incurred a poorer prognosis with a lower overall survival rate, but recent studies have challenged this notion that EBV positivity portends a poor prognosis for DLBCL, regardless of age [1,9].

The heterogeneous class of DLBCL has distinct clinical presentations, morphologies, and treatment profiles, and they’re typically diagnosed with lymph node biopsies. The gold standard for diagnosing EBV+DLBCL is by identifying EBV-encoded RNA in the nuclei of tumor cells by in situ hybridization [6]. Classically, DLBCL is diagnosed based on the following characteristics: diffuse collections of lymphoid cells; immunohistochemistry (IHC) staining of pertinent B-cell markers such as PAX5, CD19, CD20, CD22, CD30, and CD79; and presence of EBV+ RNAs [10]. 

In general, EBV+ DLBCL responds poorly to front-line treatments in comparison to their EBV-DLBCLs [10]. Current immunochemotherapy protocols include rituximab cyclophosphamide-hydroxydaunorubicin-oncovin-prednisone (R-CHOP) and rituximab cyclophosphamide-etoposide-prednisone-oncovin (R-EPOCH) therapies. Since the first introduction of rituximab, clinical outcomes of overall non-Hodgkin lymphomas have significantly improved. Due to the aggressive course of this malignancy, early detection and prompt interventions are critical for managing DLBCL early on [11]. Diverse studies have been done to address this malignancy, however, more research is needed to be done [12].

## Conclusions

Diffuse large B-cell lymphoma primarily involving the bone with no extranodal involvement is an infrequent condition. EBV accounts for less than 10% of cases as a causative agent for DLBCL. Our patient presenting with pathologic fracture and no B symptoms makes this insidious case presentation more unique. Few cases exist in the literature regarding lymphoma presenting as a fracture, thus it is important to keep into consideration the possibility of malignancy in patients that present with pathological fractures. More research is needed regarding primary bone lymphoma and its diagnostic approach and management.
